# Editorial: Medicinal plants in the treatment of gastrointestinal cancers: How can OMICS and other advanced approaches help in understanding their mechanisms of action?

**DOI:** 10.3389/fphar.2022.1063915

**Published:** 2022-11-02

**Authors:** Yibin Feng, Zhe-Sheng Chen, Hongxi Xu, Qihe Xu

**Affiliations:** ^1^ School of Chinese Medicine, The University of Hong Kong, Pok Fu Lam, Hong Kong SAR, China; ^2^ Department of Pharmaceutical Sciences, College of Pharmacy and Health Sciences, St. John’s University, Queens, NYC, United States; ^3^ School of Pharmacy, Shanghai University of Traditional Chinese Medicine, Shanghai, China; ^4^ Renal Science and Integrative Chinese Medicine Laboratory, Department of Inflammation Biology, School of Immunology & Microbial Sciences, Faculty of Life Sciences & Medicine, King’s College London, London, United Kingdom

**Keywords:** medicinal plants, gastrointestinal cancers, omics, advanced approaches, mechanisms of action

Due to the many factors involved, the treatment of cancer by Chinese medicine is very complicated: it is not only a variety of components of Chinese herbal medicines but also a complicated cancer network among tumor cells, tumor microenvironment, human interior milieu, and the environment. In addition, the interaction between the gut microbiota and the host also has a potential impact on cancer progression. The complexity of Chinese herbal medicines and the pathogenesis of cancer makes it difficult to understand the pharmacological effects of anticancer Chinese herbal medicines, especially when the treatment is in the form of a mixture of multiple compounds, such as Chinese herbal formulas.

To address such a high level of complexity, advanced approaches are needed. Thanks to the advent of systems biology and advanced OMICs techniques, including genomics, transcriptomics, proteomics, metabolomics, and bioinformatics, novel systems-based approaches have been developed and widely used in the research of Chinese medicine, leading to the rapid progress of this research field and more comprehensive understanding of the pharmacological mechanisms of Chinese herbal medicines in cancer treatment.

In this special collection, we aimed to highlight the application of OMICs and other advanced approaches in understanding the mechanisms of action of medicinal botanical drugs as a mixture of multiple components in the treatment of human diseases, especially gastrointestinal cancer. We have received 16 submissions by authors from mainland China, Macau S. A. R., and Hong Kong S. A. R, including both original articles and reviews, and seven of them are accepted for publication. Among these published studies, Yuan and others innovatively combined multiple OMICs platforms to analyze the modulation of gut flora metabolites by the polysaccharides extracted from a Chinese botanical drug *Albuca Bracteata* (Thunb.) J.C.Manning & Goldblatt in a colon cancer model. They found that the change of intestinal microbiota and therefore production of beneficial short-chain fatty acids might contribute to the anti-tumor effect of *Albuca Bracteate Polysaccharides* when used alone and in combination with the chemotherapeutic agent 5-fluorouracil Yuan et al. Another study by Shi and others performed 16S rRNA gene sequencing and metabolomics to study the mechanism of action of Siwu-Yin, a formula used in traditional Chinese medicine for the treatment of esophageal precancerous lesions and esophageal cancer. They found this formula traditionally used to replenish blood and nourish Yin improved the composition of intestinal flora by regulating the synthesis and secretion of bile acids. This action of Siwu-Yin contributed to the modification of the tumor microenvironment and inhibited the occurrence of esophageal precancerous lesions (Shi et al.). These studies have provided us insights into the application of combined OMICs technologies in the pharmacological studies of botanical drugs.

This special issue has also collected expert opinions and reviews on the application of OMICs technologies in the pharmacological studies of botanical drugs. Dai and others reviewed the state of art and provided their future perspectives on OMICs applications in studies on herbal treatment of gastrointestinal cancers. They highlighted how single and multiple OMICs approaches may facilitate unravelling signalling interaction networks and key molecular targets of botanical drugs with anti-gastrointestinal cancer potential. Their review suggested that, towards precision medicine, extensive application of high-throughput sequencing technologies to unravel tumor heterogeneity is urgently needed for stratified diagnosis of gastrointestinal cancers and better-targted herbal treatment (Dai et al.). Li and others focused on the application of OMICs technologies as useful tools for deciphering the role of botanical drugs as an adjuvant therapy, both to improve therapeutic outcomes and to reduce the side effects of conventional therapies, in the treatment of gastrointestinal cancers. They highlighted that OMICs approaches can efficiently reveal potential key molecular targets and intracellular interaction networks of herbal medicines in gastrointestinal cancer, therefore guiding future research Li et al. Guo and others particularly reviewed the application of metabolomics in investigating herbal therapies against gastrointestinal cancers by targeting metabolism. They systematically summarized the actions of herbal compounds, extracts, and formulas on the metabolism of carbohydrates, lipids, amino acids, and nucleotides, as well as on other metabolic pathways, and highlighted the usefulness of metabolomics in guiding the further development of botanical drugs as novel and efficient adjuvant therapeutics against gastrointestinal cancer (Guo et al.). In addition, *Coptidis Rhizoma (C. Rhizoma; Huanglian, in Chinese)* and Chinese Medicine-based carbon dots were introduced by He et al., and Li et al., respectively, as examples for a better understanding of the application of OMICs in pharmacological studies of herbal products. He et al. reviewed the broad pharmacological effects of *Coptidis Rhizoma (C. Rhizoma)* against cancers, highlighted recent studies on the botanical drugs, including the application of OMICS, novel drug delivery systems, combination therapy and clinical research, and provided new scientific and clinical evidence for future clinical use of the botanical drugs in treating gastrointestinal cancers (He et al.). Li et al., reviewed the progress in Chinese herbal medicines-derived carbon dots for the treatment of hemorrhagic diseases and also discussed the application of nanomaterials, OMICS and other advanced technologies in the research and development of charcoal drugs against other refractory diseases, e.g., cancers Li et al.

**FIGURE 1:**
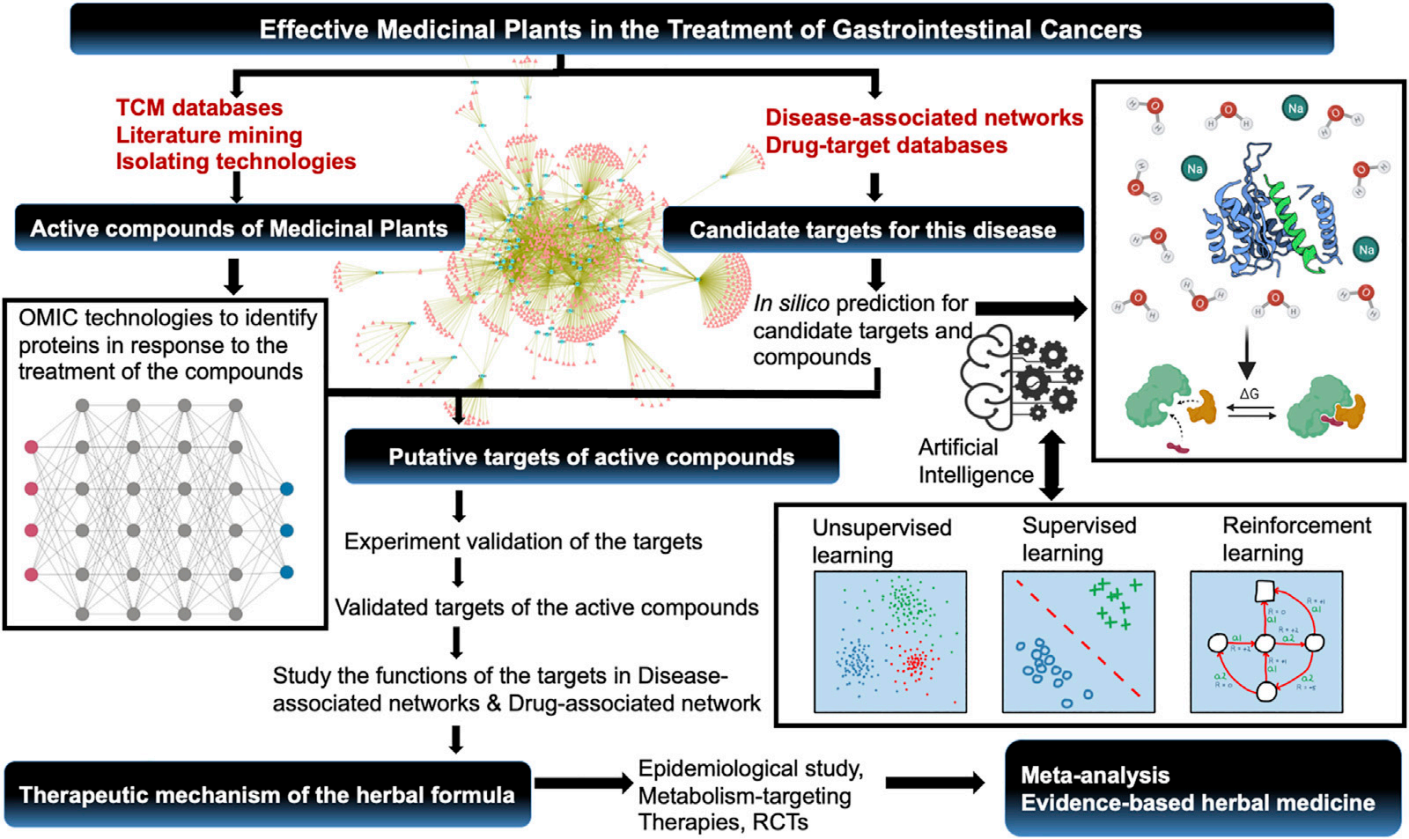
Identification of effective botanical drugs in the treatment of gastrointestinal cancers using OMICS and advanced approaches.

In conclusion, the applications of single and combined OMICs technologies, systems biology, and other advanced methodologies have been very useful in studying botanical drugs. We wish readers will enjoy reading articles in this special collection, which represent the state of the art of OMICs-based pharmacological studies of botanical drugs. As shown in Figure 1, we hope cutting-edge core technologies in multi-omics, big data and other advanced panoramic approaches will help us understand the mechanisms of action of medicinal botanical drugs and facilitate the development of safer and more efficacious preventive and therapeutic strategies.

